# Factors driving metabolic diversity in the budding yeast subphylum

**DOI:** 10.1186/s12915-018-0498-3

**Published:** 2018-03-02

**Authors:** Dana A. Opulente, Emily J. Rollinson, Cleome Bernick-Roehr, Amanda Beth Hulfachor, Antonis Rokas, Cletus P. Kurtzman, Chris Todd Hittinger

**Affiliations:** 1 Laboratory of Genetics, Genome Center of Wisconsin, Wisconsin Energy Institute, J. F. Crow Institute for the Study of Evolution, University of Wisconsin–Madison, Madison, WI 53706 USA; 2 DOE Great Lakes Bioenergy Research Center, University of Wisconsin–Madison, Madison, WI 53706 USA; 3 Applied Biomathematics, Setauket, NY 11733 USA; 4 Department of Biological Sciences, East Stroudsburg University of Pennsylvania, East Stroudsburg, PA 18301 USA; 5 Department of Biological Sciences, Vanderbilt University, Nashville, TN 37235 USA; 6 Mycotoxin Prevention and Applied Microbiology Research Unit, National Center for Agricultural Utilization Research, Agricultural Research Service, U.S. Department of Agriculture, Peoria, IL 61604 USA

## Abstract

**Background:**

Associations between traits are prevalent in nature, occurring across a diverse range of taxa and traits. Individual traits may co-evolve with one other, and these correlations can be driven by factors intrinsic or extrinsic to an organism. However, few studies, especially in microbes, have simultaneously investigated both across a broad taxonomic range. Here we quantify pairwise associations among 48 traits across 784 diverse yeast species of the ancient budding yeast subphylum Saccharomycotina, assessing the effects of phylogenetic history, genetics, and ecology.

**Results:**

We find extensive negative (traits that tend to not occur together) and positive (traits that tend to co-occur) pairwise associations among traits, as well as between traits and environments. These associations can largely be explained by the biological properties of the traits, such as overlapping biochemical pathways. The isolation environments of the yeasts explain a minor but significant component of the variance, while phylogeny (the retention of ancestral traits in descendant species) plays an even more limited role. Positive correlations are pervasive among carbon utilization traits and track with chemical structures (e.g., glucosides and sugar alcohols) and metabolic pathways, suggesting a molecular basis for the presence of suites of traits. In several cases, characterized genes from model organisms suggest that enzyme promiscuity and overlapping biochemical pathways are likely mechanisms to explain these macroevolutionary trends. Interestingly, fermentation traits are negatively correlated with the utilization of pentose sugars, which are major components of the plant biomass degraded by fungi and present major bottlenecks to the production of cellulosic biofuels. Finally, we show that mammalian pathogenic and commensal yeasts have a suite of traits that includes growth at high temperature and, surprisingly, the utilization of a narrowed panel of carbon sources.

**Conclusions:**

These results demonstrate how both intrinsic physiological factors and extrinsic ecological factors drive the distribution of traits present in diverse organisms across macroevolutionary timescales.

**Electronic supplementary material:**

The online version of this article (10.1186/s12915-018-0498-3) contains supplementary material, which is available to authorized users.

## Background

Trait correlations are widespread across life. These correlations can be intrinsic to an organism’s biology or the result of extrinsic factors. Intrinsic biological factors that can lead to associations between traits include overlapping biochemical or genetic pathways [1, 2], promiscuous enzymes [3, 4], and other forms of pleiotropy [5, 6]. Such factors can facilitate the evolution of novel traits from preexisting traits [7–9]. The environment or niche of an organism is a composite of many factors (e.g., temperature, carbon availability, and salinity), which select for suites of traits or trait syndromes that are compatible with that specific habitat or niche [10].

Trait syndromes can include a set of traits that collectively provide a fitness advantage, either additively or non-additively, in the environment and have been described for distinct groups of species that are associated with certain environments [11–14]. For example, stress-resistant syndrome is a common suite of traits that enables plants to survive in stressful environments, such as low-resource environments [15]. Stress-resistant plants tend to have lower rates of growth and photosynthesis, high root-to-shoot ratios, and additional adaptations to low-nutrient conditions. A second example is domestication syndrome, a collection of traits associated with the genetic change of an organism from a wild progenitor to a domesticated form, which is prevalent among domesticated animals [16, 17] and plants [18]. In animals, the characteristics of this syndrome can include reduction in tooth size, increased docility, reduction in brain size, and many others [17]. Alternatively, correlations between traits or suites of traits may be due to phylogeny, the retention of ancestral traits in descendant species [19, 20]. Intrinsic, extrinsic, and phylogenetic factors are not mutually exclusive, so inferences about interactions among traits must consider each of these possibilities, a task undertaken in relatively few comprehensive studies [21–25], none of which have considered microbes or metabolic traits.

The budding yeast subphylum Saccharomycotina is among the most extensively characterized higher taxonomic ranks [26]. In addition to the well-known model system *Saccharomyces cerevisiae* and the human commensal and pathogen *Candida albicans*, its more than 1000 known species share a common ancestor approximately half-a-billion years ago. These yeasts display considerable genetic, phenotypic, and ecological diversity and provide a unique opportunity to quantify trait associations and elucidate the mechanisms that drive them [27–36]. Furthermore, these yeasts have been extensively described by leading taxonomists, who have scored growth phenotypes for a large number of phenotypic traits in *The Yeasts: A Taxonomic Study* [26]. In addition to its comprehensive nature, an advantage of this phenotypic dataset is that the methods used to score yeasts for trait presence were uniform across species; therefore, there are fewer biases than would occur by combining multiple published datasets. We used this qualitative dataset of 48 traits in 784 budding yeast species to test whether physiological associations involving nitrogen and carbon source utilization (i.e., assimilation or consumption), sugar fermentation, and growth temperature traits were driven by intrinsic (biological/functional) or extrinsic (environmental) factors. We identified pervasive positive and negative correlations between traits among wild yeast species and found that the structures of metabolic networks are dominant factors that drive these associations, while environment plays an important secondary role.

## Results and Discussion

Since individual traits do not evolve independently of each other, we hypothesized that the yeast phenotypic dataset would include positive (traits that tend to co-occur) and negative (traits that tend to not occur together) associations. We quantified pairwise associations among 48 traits across 784 species. Most traits were conditional growth, such as growth in a medium with a single carbon source, and most species were represented by a single strain, the taxonomic type strain. We verified a subset of the dataset by growing 240 yeast species on four carbon sources: galactose, maltose, sucrose, and raffinose (Additional file 1: Table S1). We found that 94% of the growth results matched the data found within *The Yeasts: A Taxonomic Study*, leading us to conclude that this dataset is sufficiently accurate and reproducible.

**Fig. 1:**
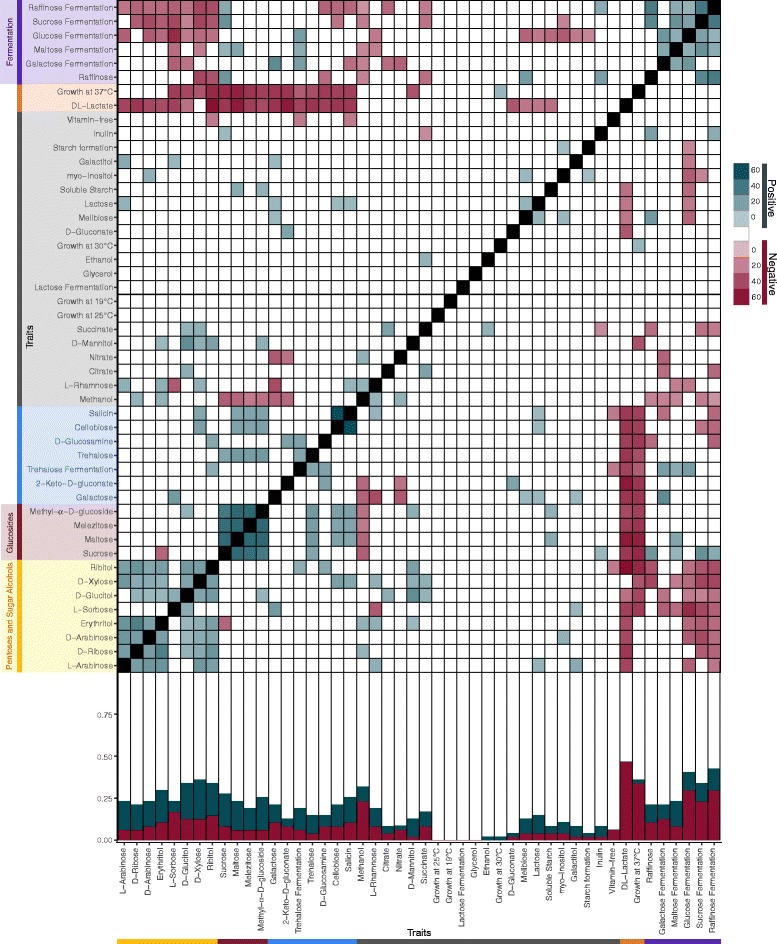
Traits showed pairwise positive and negative associations, but the numbers and strength of association varied among traits. Bottom: Stacked bar graph displaying the proportion of negative (red), positive (blue), and not significant (white) associations for all traits. Top: Heat map of pairwise associations among traits. The color of a box represents the type of association: negative (red), positive (blue), and not significant (white). The strength of the association (the difference between the observed and expected counts) is displayed by the saturation of the color. We used a hierarchical cluster analysis to determine significant trait clusters with similar pairwise trait associations (Additional file 3: Figure S1; *q* < 0.05). Selected trait clusters are represented by the colors along the left-hand side and bottom of the graph

For each trait pair, we quantified observed trait pair counts (i.e., the number of times both traits in a pairwise set were present across all species) and compared it to a distribution of counts based on randomized or permuted datasets (*n* = 10,000) to determine significance. The average of these randomized counts represents our expected value, and the difference between the observed and expected counts represents the strength of the trait pair association. We found several (*n* = 211) significantly positive (*n* = 104) and negative (*n* = 107) pairwise associations among traits (Fig. 1 and Additional file 2: Table S2). Clustering traits based on association strength revealed that traits that shared similar associations formed significant trait clusters (*p* < 0.05, Additional file 3: Figure S1), which often involved similar physiological functions (e.g., fermentation traits), chemical bonds (e.g., glucosides), and functional groups (e.g., sugar alcohols) (Additional file 2: Table S2), suggesting that the biological properties of traits could be an important factor affecting trait associations. Most negative associations occurred with sugar fermentation, growth on DL-lactate, and growth at 37 °C (88/107, Fig. 1, bottom), each of which will be highlighted below. In contrast, positive associations were broadly spread across traits and were not driven by a few individual traits (Fig. 1b, bottom).

### Intrinsic biological properties explain the largest proportion of trait variation

**Fig. 2:**
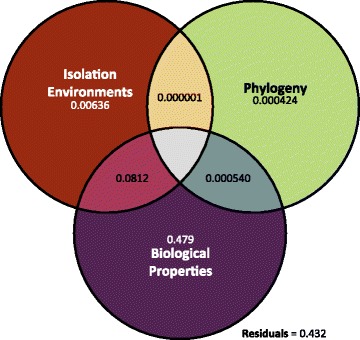
Variation in trait associations can largely be explained by biological properties. We used variance partitioning to measure the amount of variation in pairwise trait associations that could be explained by biological properties (purple), isolation environments (red), and phylogeny (green). Each circle represents a single factor, while the overlap among circles represents interactions among factors (e.g., the interaction between biological properties and isolation environments is represented by the pink area). The values provided in each area represent the amount each factor contributes to the variation. In total, the factors represented here explain 0.568 (or 56.8%) of the variance in trait associations. The residuals represent the proportion of the variance that is unexplained

Multiple factors could conceivably contribute to the positive and negative trait associations we detected. We quantified the extent to which phylogeny, biological properties, and isolation environments contributed to the variance of trait associations [37]. This variance decomposition showed that biological properties (i.e., the functional attributes and structures of molecules consumed) largely drove the variation in trait associations. Specifically, 47.9% of the variance was explained by biological properties alone (*R*^2^ = 0.479, Fig. 2), while phylogeny and isolation environments explained only 0.0424% (*R*^2^ = 0.000424, Fig. 2) and 0.636% (*R*^2^ = 0.00636, Fig. 2) of the variance on their own, respectively. In some instances, multiple factors jointly explained the variance of trait associations. The largest proportion of these complex factors occurred between biological properties and isolation environments, which together explained 8.12% of the variance (*R*^2^ = 0.0812, Fig. 2). Only 0.0541% of the variance could be explained by both phylogeny and isolation environments or by both phylogeny and biological properties. In total, some combination of these three factors explained 56.8% of the variance in trait associations, leaving 43.2% unexplained**.** These results show that the vast majority (98%) of the explained variation among trait associations involves biological properties intrinsic to the organism, while isolation environments play an important secondary role, often in conjunction with biological properties.

### Limited effects of evolutionary history

Despite the limited amount of variance explained by phylogeny in our full dataset, we tested individual traits for a significant phylogenetic signal by calculating *D* across individual traits using a phylogeny of 561 species [38]. *D* is a measure of the character dispersion of binary traits and is similar to the more commonly used Blomberg’s *K* and Pagel’s ***λ*** statistics, which are used for quantitative data. Negative *D* values represent phylogenetic clustering, whereas values greater than 1 indicate phylogenetic over-dispersion. When *D* is not significantly different from 0, the trait is evolving according to a Brownian motion process that tracks phylogeny. When *D* does not significantly differ from 1, that trait is randomly distributed across the phylogeny. Although we detected some phylogenetic signal for most individual traits (i.e., *D* significantly differed from 1 for 47/48 traits), these analyses also rejected simple Brownian motion for the majority of traits (i.e., *D* significantly differed from 0 for 31/48 traits), suggesting that factors other than phylogeny predominate (Additional file 4: Table S3). Along with the variance decomposition (Fig. 2), these results suggest that, although phylogenic history is significantly associated with some individual traits, it is not the major driver of the observed trait associations.

### Environmental factors help drive trait associations

To test the role of isolation environments in trait associations further, we compiled and categorized the environments from which each species had been isolated from *The Yeasts: A Taxonomic Study* and quantified the association between isolation environments and traits using the same methods to quantify trait associations (Additional file 5: Table S4). Yeasts have been isolated from many environments, including insects, plants, and food (Additional file 6: Figure S2). These environments were scored hierarchically, ranging from general categories (e.g., insects) to specific categories (e.g., ants). The broader categories increased the sample sizes available for statistical analyses, but they may also aggregate cryptic ecologies.

**Fig. 3:**
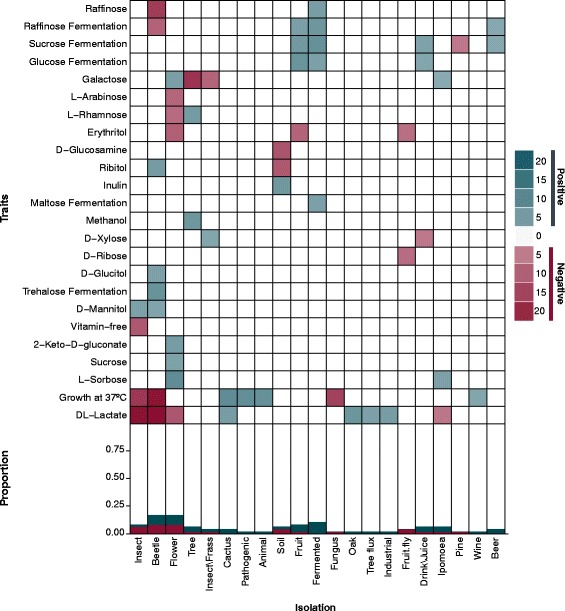
Traits are positively and negatively associated with a subset of isolation environments (*q* < 0.05). The numbers and strengths of these associations varied among traits and environments. Bottom: Stacked bar graph displaying the proportion of negative (red), positive (blue), and not significant (white) associations for each isolation environment with at least one significant association. Top: Heat map of pairwise associations among traits and isolation environments. The color of a box represents the type of association: negative (red), positive (blue), and not significant (white). The strength of the association (the difference between observed and expected trait counts) is displayed by the saturation of the color

Isolation environments differed by the numbers and types of traits positively and negatively associated with them (*p* < 0.05, Fig. 3 and Additional file 7: Table S5). The strengths of these associations also varied among traits and environments, partly due to differences in power. Significant associations between traits and environments were generally concordant with known ecologies. For example, glucose and sucrose fermentation were positively associated with fruit, fermented substrates, and drinks or juice. These associations were not driven solely by the genus *Saccharomyces* and included many non-*Saccharomyces* yeasts known to be important for fermentation and spoilage of drinks, including yeasts that have been commercialized as oenological starter cultures due to their fermentation capabilities and their contributions to the chemical compositions of wines (e.g., *Hanseniaspora uvarum*, *Torulaspora delbrueckii*, *Metschnikowia pulcherrima,* and *Lachancea thermotolerans* [39]). *H. uvarum* and *T. delbrueckii* have also both been shown to be involved in the early stages of spontaneous fermentation of grapes, demonstrating that these yeasts ferment fruits in their natural environments [39, 40].

Growth at 37 °C was positively associated with isolation from cacti (*p* < 0.001), which prefer warmer climates. Yeast communities isolated from the family Cactaceae were previously associated with growth at high temperatures, although species identifications were not possible in this classic 1986 study [41]. Our analyses extend this pre-molecular research to specific yeast taxa (e.g., *Pichia cactophila and Clavispora opuntiae*), provide further statistical support for this association, and highlight its relative importance in a more comprehensive picture of yeast ecology. Similarly, our analyses statistically supported the previously hypothesized positive associations between growth on DL-lactate and isolation from oaks and cacti [41].

As expected, we also found growth at 37 °C was also positively associated with yeasts being classified as pathogenic (*p* = 0.0047). This oft-stated association [26, 28] stems from the need to survive elevated temperatures within endothermic hosts, but to our knowledge, it has never been formally tested across a broad taxonomic scale. In contrast, growth at 37 °C was negatively associated with isolation from insects (*p* = 0.03) and fungi (*p* < 0.001), which are not endothermic. More intriguingly, the endothermic pathogenic/commensal lifestyle could help explain the unexpected negative associations that we observed between growth at 37 °C and the ability to utilize a variety of carbon sources (15/33 carbon sources, Table 1) because emerging evidence suggests that carbon starvation is frequently encountered by yeasts growing in mammalian hosts [42–44].

**Table 1:** Utilization of diverse carbon sources is negatively associated with growth at high temperatures

Utilization	Observed	Expected	*p*_*adj*_	Difference
Sucrose	428.000	374.15	<0.0001	53.8478
Galactose	445.000	396.3	<0.0001	48.7038
Trehalose	418.000	377.93	0.007	40.0716
Maltose	424.000	366.55	<0.0001	57.4544
Melezitose	402.000	352.02	<0.0001	49.9786
Methyl-α-D-glucoside	386.000	340.78	<0.0001	45.2166
Cellobiose	416.000	370.55	<0.0001	45.4488
Salicin	416.000	371.1	<0.0001	44.896
L-sorbose	429.000	382.19	<0.0001	46.8062
D-xylose	412.000	375.23	0.010	36.769
Ribitol	430.000	375.53	<0.0001	54.4678
D-mannitol	438.000	399.61	<0.0001	38.386
D-glucitol	447.000	404.41	<0.0001	42.5884
D-glucosamine	402.000	354.46	<0.0001	47.5448
2-keto-D-gluconate	413.000	363.27	0.002	49.7272

The observed data are a count of when one trait (e.g., the carbon utilization trait or growth at 37 °C) is present, while the other trait is absent. The expected value is the average count of the presence of either the carbon utilization trait of interest or growth at 37 °C and the absence of the other trait across 10,000 permutations. The difference is the observed minus the expected columns. We corrected for multiple tests across associations with the Benjamini–Hochberg correction (*q* < 0.05 shown, all data in Additional file 2: Table S2), which is shown in the column *p*_adj_

**Fig. 4:**
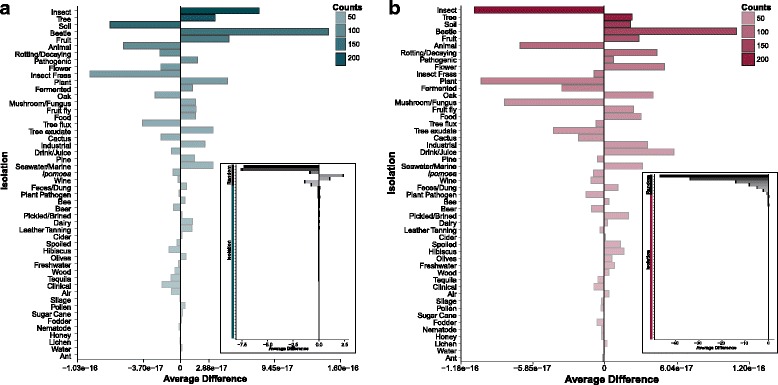
Isolation environments contribute to positive and negative associations among traits. We calculated the deviations for our observed data for both **a** positive and **b** negative associations. All of the deviations for the observed data were close to zero, suggesting that isolation environments contribute to the trait associations. The saturation of the bars represents sample sizes. In the insets, we removed the effect of the environment by randomly sampling species without regard to their environment for multiple sample sizes (4 ≤ *n* ≤ 217) and reproduced the environment data on the same scale for contrast (isolation versus random). Note that removing the effect of environment led to significant deviations from expectations for both positive (inset in **a**) and negative (inset in **b**) associations

The ability to survive in certain habitats requires suites of traits, while other traits are expendable [10, 15]; therefore, both negative and positive trait associations could be affected by extrinsic factors related to isolation environments. To explore the extent to which extrinsic factors contributed to trait associations further, we tested whether the number of trait associations we saw in each specific environment was more than that expected by chance. If there were more significant observed trait associations (negative or positive) within a given environment than expected, we would conclude that the specific isolation environment tested was not important to the significant trait associations. Instead, for all isolation environments, we found no significant differences between the observed and expected associations for both the positive (Fig. 4a) and negative (Fig. 4b) associations. In contrast, when we removed the effects of the environment by drawing species from random environments and repeating our analyses as a control, we found significant deviations from the number of positive and negative associations. These results demonstrate that the environment is an important factor in explaining observed trait associations, even if it may often be acting in concert with intrinsic biological factors.

### Networks of intrinsic biological factors affecting traits

**Fig. 5:**
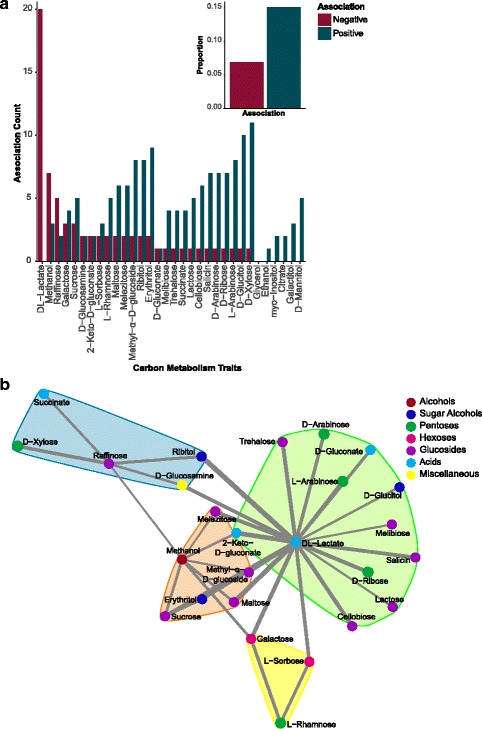
Significant associations among carbon metabolism traits were biased toward positive associations. **a** Bar graph displaying the total number of positive (blue) and negative (red) associations for each carbon source (Additional file 5: Table S4). **b** Negative association network between carbon utilization traits. The width of the edge between nodes represents the strength of the association between carbon sources. Node color represents similar molecular structures among compounds, and shading behind nodes represents significant communities within the network. Note that, among negative associations, the majority (79%) of significant associations were with DL-lactate and methanol

Given the amount of the variance in trait associations explained by biological properties, we examined the role of biological factors in more detail. In particular, promiscuous enzymes and pathway overlap are major features of yeast carbon metabolism that could potentially underlie many of the scored traits and the significant trait clusters (*p* < 0.05, Fig. 1 and Additional file 3: Figure S1). Shared enzymes and pathways are expected to lead primarily to positive associations, and indeed, we found an enrichment for positive associations relative to negative associations among carbon source utilization traits (*p* = 7.67 × 10^− 6^). Across trait pairs (*n* = 496), 75 pairs were significantly positively associated, while only 34 were significantly negatively associated (Fig. 5a and Additional file 8: Table S6). Moreover, DL-lactate (*n* = 20) and methanol (*n* = 7) resulted in 79% of all negative associations with other carbon sources, and most carbon sources were not negatively associated with any beyond these two (Fig. 5b).

**Fig. 6:**
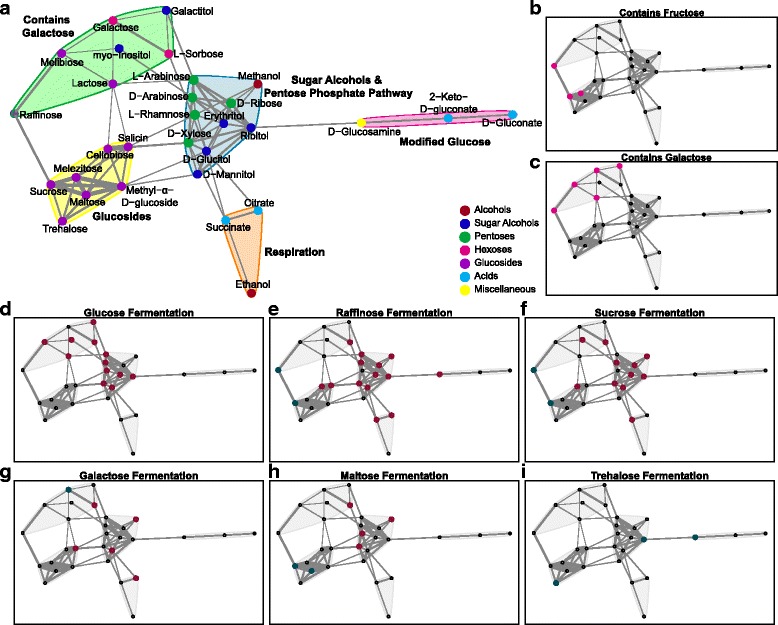
Carbon source utilization trait associations form communities within the network that contain traits with similar structures and/or properties. **a** Network of positive associations among carbon utilization traits. The width of the edge between nodes represents the strength of the association between carbon sources. Node color represents similar molecular structures among compounds, and shading behind nodes represents significant communities within the network. Each subnetwork is labeled with a description of the biochemical structures or pathways captured by that subnetwork. **b**, **c** Reproduction of network highlighting compounds containing specific monosaccharide moieties. **d**–**i** Reproduction of network highlighting negative (red) and positive (blue) associations with the fermentation of specific sugars. Note that fermentation of a specific sugar is always positively associated with utilization of that sugar because utilization is a prerequisite for fermentation; in some cases, related sugars are also positively correlated. Trehalose fermentation may be an exception to the general trend of negative associations with other carbon sources because many yeasts synthesize trehalose internally [74]

To explore the role and cause of trait associations among carbon utilization traits further, we generated a network of all significant positive associations (Fig. 6). We detected five distinct communities or subnetworks within the complete network using the Clauset–Newman–Moore algorithm, a modularity maximization method that detects communities by searching for subdivisions with high modularity [45]. Many of the carbon sources within each subnetwork shared similar molecular structures and functional properties (Additional file 9: Table S7). For example, we detected a subnetwork that consisted exclusively of *Glucosides*, which all contain a glucose moiety linked to other chemical groups (*p* = 3.57 × 10^− 5^, Fig. 6a). Similar types of glucosidic bonds are often cleaved by promiscuous enzymes [46], and our findings suggest that these biochemical properties at least partly explain trait associations across macroevolutionary timescales. The *Contains Galactose* subnetwork (*p* = 0.0001, Fig. 6a) included all three glucosides not found in the *Glucosides* subnetwork (the galactosides melibiose, raffinose, and lactose, Fig. 6c), as well as galactose and its sugar alcohol, galactitol. The two trisaccharides in the dataset even had significant edges connecting them to their constituent disaccharide moieties (raffinose to melibiose and sucrose, as well as melezitose to sucrose, Fig. 6b).

Finally, the *Sugar Alcohols & Pentose Phosphate Pathway* subnetwork was enriched (*p* = 1.81 × 10^− 5^) for pentoses and sugar alcohols; 30 interactions occurred among nodes within the subnetwork, compared to 13 outside the subnetwork. Sugar alcohols accumulate as intermediate products during pentose utilization in many microbes [47], including yeasts [48, 49], suggesting that overlapping biochemical pathways may explain the association between the utilization of these two types of carbon sources. Strikingly, the ability to ferment various sugars was negatively associated with the ability to utilize sugar alcohols and pentoses (9 out of 11 pentoses/sugar alcohols, *p* = 0.006, Fig. 1 and Fig. 6d–i). Efficient fermentation of pentoses, such as xylose and arabinose, is one of the central challenges of bioenergy research, and this broad negative association across yeasts further underscores that only a handful of yeast species have naturally evolved pentose fermentation [50–53].

### Biochemical pathways associated with intrinsic biological factors

The enrichment for positive associations among carbon sources and the communities of carbon sources with similar properties and structures suggests that the ability to utilize one carbon source can increase the potential to utilize similar carbon sources. These findings provide an empirical and evolutionary underpinning for a theoretical model of bacterial central carbon metabolism, which proposed that metabolisms viable on one carbon source can be preadapted to multiple other carbon sources as a result of shared pathways [7]. Furthermore, the enrichment for specific molecular properties, chemical structures, and pathways within communities suggests an underlying genetic and biochemical basis for the suite of carbon metabolism traits present within a given yeast species [54].

To determine whether the observed macroevolutionary patterns of metabolic trait associations could indeed be explained by the shared genetic and biochemical pathways, we measured the extent to which the presence of several well-characterized genes from model organisms could explain growth on various carbon sources. We focused on 79 species with extensive trait information, fully sequenced genomes, and a well-resolved phylogeny [55]. For traits, we focused on two significant communities within the positive carbon metabolism network (Fig. 6) and searched the genomes for homologs of genes known to enable the utilization of those carbon sources in model organisms (Additional file 10: Table S8).

For the *Contains Galactose* community, the *GAL1*, *GAL7*, and *GAL10* genes, which encode the enzymes required for galactose utilization, were positively associated with galactose utilization (*p* < 0.05, Additional file 10: Table S8 and Additional file 11: Figure S3a) [56]. To test whether interacting pathways could indeed explain some of the significant edges within communities, we also tested whether galactose utilization was positively associated with the presence of *MEL1* and *LAC12*, which encode galactosidases that are required for melibiose and lactose utilization, respectively. Indeed, even though *MEL1* and *LAC12* are not required for galactose utilization, we found significant associations across macroevolutionary timescales (*p* < 0.05, Additional file 11: Figure S3a, Additional file 12: Table S9).

The *Glucosides* community was rich in individual genes (e.g., *MAL11* and *IMA5*) associated with growth on multiple carbon sources, including maltose, melezitose, and sucrose (*p* < 0.05, Additional file 10: Table S8 and Additional file 11: Figure S3b). These results suggest that these genes are pleiotropic and could be responsible for the utilization of multiple carbon sources, in line with an extensive body of research in *Saccharomyces* showing enzyme promiscuity [57], as well as with the demonstration that the deletion of *MAL1* and *MAL2* in *Ogataea polymorpha* prevented this distantly related yeast from growing on multiple carbon sources, including maltose, sucrose, and melezitose [58]. Our comprehensive analyses suggest that the established mechanisms from these model systems are general and further show how these promiscuous enzymes have led to positive trait associations across a broad taxonomic range.

Finally, we quantified whether genes that are involved in the metabolism of carbon sources within each community were more likely to co-occur. We found a significant difference in the frequency of co-occurrence among genes that were from the same community (81.1%) versus when they were from two different communities (60.2%) (*Χ*^2^ = 7.98, *p* = 0.005, Additional file 11: Figure S3c). In other words, genes involved in the utilization of positively associated carbon sources co-occurred more frequently than those of randomly associated carbon sources. The significant co-occurrence of genes associated with the utilization of positively associated carbon sources provides further support that intrinsic biological properties contribute to positive trait associations.

## Conclusions

As seen in non-microbial taxonomic groups [22, 25], trait pairs in the budding yeast subphylum show significant positive and negative associations of varying strengths. Positive associations are particularly common among carbon metabolism traits, relative to negative associations, especially those involving compounds with similar molecular structures and pathways. These correlations suggest that the ability to metabolize individual carbon sources can increase the potential to utilize additional chemically related carbon sources. Among negative associations, there are no absolute exclusions where the presence of one trait is perfectly correlated with the absence of another, suggesting that any subtle trade-offs that may occur can be overcome across macroevolutionary timescales. One caveat to our study is that these data are a qualitative measure of growth. Measuring correlations between quantitative growth parameters, such as lag, growth rate, and saturation, may reveal trade-offs or interactions among traits that cannot be seen with the current data. An interesting avenue of future research would be to measure quantitatively the growth parameters of all species and look for positive and negative associations.

The presence of negative associations could conceivably be explained by intrinsic biological factors leading to trade-offs or due to extrinsic factors. For example, highly fermentative yeasts might be intrinsically poor pentose fermenters due to a metabolic trade-off, possibly explaining the challenges encountered by biofuel researchers [59–61]. Alternatively, negative associations could also be explained by extrinsic factors, such as adaptation to the environment or an ecological niche. Under this second model, environments that select for robust pentose metabolism might not typically favor highly fermentative species. Similarly, there could be a trade-off between the utilization of a broad array of carbon sources and growth at high temperatures, as suggested by research demonstrating impaired growth on different carbon sources at 37 °C in *Candida albicans* [43]. Alternatively, yeasts may simply encounter a more limited range of carbon sources within mammalian and endothermic hosts [44], resulting in the negative association between carbon utilization breadth and growth at 37 °C, perhaps through the neutral loss of metabolic pathways [56]. The intrinsic explanations posit limitations to adaptability imposed by the metabolic network, while the extrinsic explanations propose a dominant role for the ecological niche in determining which traits are retained or acquired. An interesting future research avenue will be to determine whether similar patterns are observed in other large clades of diverse eukaryotic microbes, or perhaps even in bacteria where widespread horizontal gene transfer may further diminish the role of phylogeny.

By accounting for both intrinsic and extrinsic factors, our results demonstrate that both genetics and environment contribute to trait associations. Indeed, these factors likely interact to create a positive feedback loop, in which an organism’s genes ultimately underlie the traits needed for survival in an environment, and the environment then puts additional selective pressure on those genes, leading to their retention or further adaptation [62]. Together, these forces shape the traits present in an organism, strengthen correlations between those traits among organisms, and select for common suites of traits or trait syndromes in diverse clades.

## Methods

### Species

We examined trait correlations in 784 Saccharomycotina species [26] from 50 different isolation environments. We quantified the phylogenetic signal across our trait data for 578 species and curated isolation data for 831 yeast species.

### Trait data

Qualitative trait data for 75 traits were curated from *The Yeasts: A Taxonomic Study* [26] (Additional file 13: Table S10). The trait data are largely based on the type strain of a species; however, in some cases, multiple strains were assessed for a species and condensed into one value by the taxonomist. Traits were scored by multiple taxonomists, but they used standardized protocols to limit the biases of multiple techniques [26]. When multiple strains were qualitatively assessed for a species and only some strains grew, the trait was scored as “variable.” In the dataset, 6% of the trait matrix was marked as variable. The occurrence of two traits being variable within the matrix was less than 1%; therefore, all traits scored as variable were considered positive and scored as a 1 (Additional file 14: Table S11)**.** Trait data were not available for every trait for every species; therefore, any trait that was evaluated for presence or absence in fewer than 80% of the species was removed from all analyses. Species that were lacking trait data for more than 80% of the traits considered in our study were also removed from analyses, leaving 784 species and 48 traits with sufficient data.

### Growth validation

We validated the growth of 240 yeast species in our dataset on four carbon sources, galactose, maltose, sucrose, and raffinose. We inoculated the type strains of all species in yeast extract peptone dextrose and allowed them to grow for 3 days. After the initial inoculation, the cultures were arrayed into a 96-well plate and a pinner was used to inoculate a 96-well plate containing minimal media plus 2% sugar. The cultures grew for a week and were then scored for growth. We repeated the growth validation experiment three times. A species was scored as growing on a carbon source if it grew at least two or three times. After blindly scoring the yeasts for growth, we compared the results to the trait data from *The Yeasts: A Taxonomic Study*.

### Imputations

Since the trait data contained missing values, to determine the best way to handle these values, we tested three methods: (1) all missing values were set to 0, (2) all missing values were set to 1, and (3) missing values would be either 1 or 0. Overall, the third method correctly predicted trait presence or absence 79% of the time, while the other methods predicted trait presence or absence correctly less often (Additional file 15: Table S12). Trait associations were generally insensitive to how missing values were handled, and the methods agreed on trait associations 99% of the time. Therefore, we performed the remaining analyses using the third method (imputation).

The missing data were imputed and replaced with a value of 1 or 0 by calculating the probability of a value being 0 in a species, *P(s)*, and trait, *P(t)*, respectively:

$$ {\displaystyle \begin{array}{l}P(s)=\frac{n_0}{n_s},\\ {}P(t)=\frac{n_0}{n_t},\end{array}} $$

where *n*_0_ is the number of 0 values found for that species or trait, and *n*_*s*_ and *n*_*t*_ are the total number of data points for that species and trait, respectively. The proportion of 0 values was also calculated for the total matrix, *T(z)*:

$$ T(z)=\frac{n_0}{n_r\times {n}_c}, $$

where *n*_*r*_ and *n*_*c*_, are the total number of rows and columns in the matrix, respectively. These values were then multiplied to determine the probability that the missing data for that cell would be 0, *P(0)*:

$$ P(0)=T(z)\times \left(P(s)\times P(t)\right). $$

When *P(0)* was greater than 0.5, the missing value was set to 0, and when it was less than 0.5 the missing value was set to 1. This imputation method quantitatively accounts for the observation that some traits are more common than others, while outperforming approaches encoding all missing values as 0 or 1.

### Associations

We permuted the trait presence/absence matrix (*n* = 10,000 permutations) using a swap algorithm to perform the permutations (*n* = 1000 swaps per permutation) and maintain row and column sums from the original matrix. Permutations were performed using the R package *picante* (v. 1.6–2) [63]. To determine whether traits were positively or negatively associated, for each trait pair, we counted the number of times that both traits were observed (1,1) and the number of times only a single trait was present (1,0 or 0,1) across species, respectively:

$$ {\displaystyle \begin{array}{l}{\mathrm{Positive}}_{\mathrm{obs}}={\mathrm{obs}}_{\left( 1, 1\right)},\\ {}{\mathrm{Negative}}_{\mathrm{obs}}={\mathrm{obs}}_{\left(1,0\right)}+{\mathrm{obs}}_{\left(0,1\right)}.\end{array}} $$

These values were also calculated for the permuted data, and the expected trait pair counts were determined by calculating the mean of those permuted observations:

$$ {\displaystyle \begin{array}{l}\overline{{\mathrm{Pos}}_{\mathrm{exp}}}=\frac{\sum {\exp}_{\left( 1, 1\right)}}{10, 000},\\ {}\overline{{\mathrm{Neg}}_{\mathrm{exp}}}=\frac{\sum {\exp}_{\left(0,1\right)}+{\exp}_{\left(1,0\right)}}{10,000}.\end{array}} $$

The strength of each association (*Str*) was calculated by subtracting the observed and average accepted values for the positive and negative associations, respectively:

$$ {\displaystyle \begin{array}{l}{\mathrm{Str}}_{\mathrm{pos}}=\left|{\mathrm{Pos}\mathrm{itive}}_{\mathrm{obs}}-\overline{{\mathrm{Pos}}_{\mathrm{exp}}}\right|,\\ {}{\mathrm{Str}}_{\mathrm{neg}}=\left|{\mathrm{Neg}\mathrm{ative}}_{\mathrm{obs}}-\overline{{\mathrm{Neg}}_{\mathrm{exp}}}\right|.\end{array}} $$

We calculated the binomial confidence intervals to determined significant associations using the R package *Hmisc* (v. 4.0–0) [64] and corrected for multiple tests across associations with the Benjamini–Hochberg correction. *p*_*adj*._ or *q* < 0.05 was accepted as significant, and decreasing the significance cutoff to *q* < 0.01 did not affect our general conclusions. For all significant associations, we reported the observed [the count of either (1,1) or (1,0 or 0,1) within the actual dataset] and expected values [the mean of either (1,1) or (1,0 or 0,1) for the randomized dataset (*n* = 10,000)] for that association (Additional file 1: Table S1). Since negative and positive associations were calculated separately, if an association was not significant for either a positive or negative association, we reported the observed count for the highest difference between observed and expected (Additional file 1: Table S1). The same method was applied to determine trait isolation associations.

### Significant trait clusters

We determined whether traits showed similar patterns of associations via cluster analysis. We calculated a dissimilarity matrix, using a Euclidean distance, for all trait pairs using the difference between observed and expected association values for both the positive and negative associations. All associations that were not statistically significant were set to a value of 0. Traits were clustered using Ward’s method in the R package *pvclust* (v. 2.0–0) [65].

### Variance partitioning of trait associations

Adjusted bi-multivariate statistics (${R}_a^2$) were computed using the varpar() function in the R package *vegan* (v. 2.4–3). This statistic estimates the contributions of the independent variables (phylogeny, biological properties, and isolation environments) to the response variable (trait association). Three matrices were used for the independent variables. For the phylogenetic matrix, D1/D2 sequences from the *rDNA* locus for 578 Saccharomycotina species in our association analysis and the outgroup basidiomycete *Cryptococcus neoformans* were used to construct a phylogenetic tree of the subphylum Saccharomycotina [26, 66, 67]. All sequences were aligned using MAFFT (v. 7.305) [68, 69]. RAxML-HPC BlackBox (v. 8.2.9) was applied to build the phylogenetic tree under the GTRCAT model for nucleotide sequences; 1000 bootstrap replicates were used to assess the reliability of internal branches (Additional file 16: Table S13) [69, 70].

The inferred maximum likelihood tree was then used to make a matrix using the cophenetic.phylo() function in the R package *ape* (v. 4.1). The biological properties matrix calculated the numbers of each type of carbon source (defined in Additional file 9: Table S7) utilized by a species (e.g., the number of hexoses utilized by *S. cerevisiae*), and the isolation environments matrix was a binary matrix consisting of all species and whether or not they were isolated from an environment. The response variable was a trait association matrix that consisted of whether two traits (e.g., sucrose and maltose utilization) were both present (1) or one trait was present while the other was absent (0) in a species.

### Phylogenetic signal of traits

The D1/D2 maximum likelihood tree was used to detect the phylogenetic signal in our trait data. We determined whether there was a phylogenetic signal for individual traits by calculating *D*, a measure of dispersion for binary traits [38], and testing for a significant departure from both random associations (*D* = 1) and the clumping expected under a Brownian model of evolution (*D* = 0). It was calculated using the phylo.d function in the R package caper (v.0.5.2) [71]. We used 1000 permutations to detect whether *D* was significantly different from random associations and clumping.

### Isolation environments

Isolation environments were manually scored and curated from the “Ecology” section for each species available in *The Yeasts: A Taxonomic Study* [26]. Isolation environments were classified into specific isolation conditions (e.g., oak, ant, and beer) and broad isolation conditions (e.g., tree, insect, and fermentation). Each isolation environment was scored as a 1 or 0 to represent species presence and absence in each environment (Additional file 5: Table S4). If the isolation environment of a species was unknown, it was classified as unknown. Analyses were performed for isolation environments that contained four or more species.

### Quantifying direct and indirect associations

To determine whether the negative and positive associations among traits were indirectly caused by the environment, we calculated the average difference between the observed and expected numbers of trait associations for each environment using a co-occurrence matrix [72]. Any deviations from 0 would suggest the trait associations observed were driven by something other than the isolation environment. We removed the effects of the environment by randomly drawing species, regardless of their isolation environment, for a range of sample sizes (*n* = 4, 11, 26, 47, 76, 147, 217). For each sample size, we randomly drew that number of species from our trait data set and ran the analysis described above. We did this for each sample size 1000 times and calculated the average difference for each sample size.

### Carbon metabolism network analyses

To quantify whether there was an enrichment of positive associations in our association data, the data were limited to carbon metabolism traits that had at least one significant association, and we used a two-sided Fisher’s exact test. Positive and negative association networks were created in the R package *igraph* (v. 1.0.1) [73], and carbon trait communities in these networks were determined through the Clauset–Newman–Moore algorithm (*fast.greedy* community), an algorithm that maximizes modularity. We determined whether there was enrichment for specific molecular properties or functions within each subnetwork using two-sided Fisher’s exact tests.

### Gene carbon analysis

Gene presence was detected using TBLASTX and BLASTN searches using query sequences [53] from the characterized pathways in model organisms (e.g., *S. cerevisiae*) versus 79 previously curated genome assemblies [55], using an *e* value cutoff of 10^−10^. We also collapsed the *MAL12* and *IMA1–4* genes into a single *IMA/MAL* group since these genes are closely related paralogs [57]. For each carbon source, we quantified how often each gene was present and there was growth on the carbon source, as well as the sum of how often a gene was absent and there was growth plus how often a gene was present and there was no growth. We used a *Χ*^2^ test to detect associations between growth and gene presence and corrected for multiple tests across associations with the Benjamini–Hochberg correction. We also tested whether genes of positively associated traits co-occurred more frequently than those of traits that showed random associations using a test for equal proportions. We included two groups of genes in our analyses; the first group of genes (*Contains Galactose*) consisted of *GAL1*, *GAL7*, *GAL10*, *LAC12*, and *MEL1.* The second group (*Glucosides*) comprised *MAL11*, *MAL13*, *MAL6*, the *IMA*/*MAL*-collapsed genes, *IMA5*, and *SUC2*. We tested whether genes within each group co-occurred more frequently than genes that were from the two different groups. All statistical analyses were done in R.

## Additional files

Additional file 1: **Table S1.** Growth validation of 240 yeasts on four carbon sources. Growth on a carbon source was scored as 1, while no growth was scored as 0. Cases where our data did not match *The Yeasts: A Taxonomic Study* are indicated with an X. A summary, including the counts of mismatched data, proportion of mismatches, and percentage correct, is given below each column. (XLSX 17 kb)

Additional file 2: **Table S2.** All pairwise trait associations. The “Direction” column indicates whether a trait association was classified as a positive, negative, or random association. The expected value is the average value from the randomized data (*n* = 10,000). (XLSX 75 kb)

Additional file 3: **Figure S1.** Hierarchical cluster analysis of traits, using similarities among trait associations. The different colors represent selected significant clusters. Biologically meaningful clusters were named accordingly. Note that the strengths of the positive and negative associations with fermentation and with growth at high temperatures (37 °C) lead to clustering, which, in some cases, obscures the subtler associations among carbon source utilization traits that are readily observed in the network analysis presented in Fig. 6. (PDF 285 kb)

Additional file 4: **Table S3.** Over 50% of individual traits do not evolve under a Brownian model of evolution. To test for a phylogenetic signal in our data, we calculated *D*, a measure of dispersion used for binary traits that tests for a significant departure from both random associations (*D* = 1) and the clumping expected under a Brownian model of evolution (*D* = 0). Values that significantly differ from 0 suggest a trait is not evolving under Brownian evolution. The function calculates randomized data sets (*n* = 1000) for both departures from random distributions and patterns expected under a Brownian model of evolution. To test for phylogenetic randomness, the function randomly shuffles trait values relative to the tips of the phylogeny. To test for evolution under a Brownian model, the function evolves a continuous trait along the phylogeny under a Brownian process, and then it is converted to a binary trait using a threshold that reproduces the relative prevalence of the observed trait. (XLSX 11 kb)

Additional file 5: **Table S4.** Species isolation environment matrix. A value of 1 means the species was isolated from that environment, while a value of 0 means the species was not isolated from that environment. (XLSX 94 kb)

Additional file 6: **Figure S2.** Bar graph of number of species found in each isolation environment. (PDF 185 kb)

Additional file 7: **Table S5.** Pairwise association quantifications among traits and isolation environments. The “Direction” column indicates whether an isolation/trait association was classified as positive, negative, or random. The expected value is the average value from the randomized data (*n* = 10,000) (XLSX 111 kb)

Additional file 8: **Table S6.** Number of positive, negative, and random associations among carbon utilization traits. (XLSX 10 kb)

Additional file 9: **Table S7.** Assigned functional categories used statistically to analyze the similarities between carbon sources. (XLSX 11 kb)

Additional file 10: **Table S8.** Gene and trait presence for 79 species with curated genome sequences [55]. Gene presence was detected using TBLASTX and BLASTN searches using query sequences from the characterized pathways in model organisms (e.g., *S. cerevisiae*) versus the curated genome assemblies [55], using an *e* value cutoff of 10^− 10^. (XLSX 15 kb)

Additional file 11: **Figure S3.** Genes show pleiotropic functions for carbon utilization. We determined gene presence for 79 species for which we had trait data and curated genome sequences [55]. We quantified associations with gene presence and growth on two sets of carbon sources that show similar trait associations in Fig. 6. **a** Bar graph of growth on carbon sources (*Contains Galactose*) when genes are present (gray) or absent (white). Significant associations are denoted with an asterisk. It is well established that galactose utilization is associated with the presence of the *GAL1*, *GAL7*, and *GAL10* genes [56]; this result serves as a control for our BLAST cutoffs. *MEL1* and *LAC12* are galactosidases that cleave the disaccharides melibiose and lactose, respectively, and were also significantly associated with galactose utilization across macroevolutionary timescales. The melibiose and lactose utilization data are consistent (but not significant) with this trend; however, there are few species that can use them. **b** Bar graph of growth on carbon sources (*Glucosides*) when associated genes are present (gray) or absent (white). We found significant associations (asterisk) between glucoside utilization genes and multiple carbon sources. These results suggest that pleiotropic genes could be responsible for the utilization of multiple carbon sources. **c** We quantified whether genes associated with traits that show positive trait associations co-occur more frequently than those that show random associations. Genes associated with positively associated traits (e.g., from **a**, *GAL1, GAL7, GAL10, MEL1,* and *LAC12* comprise one set of genes, and from **b**, *IMA5*¸ *IMA/MAL* collapsed genes, *MAL11*, *MAL13*, *MAL6*, and *SUC2* make up the second set) co-occurred 81.1% of the time (red), while genes associated with randomly associated traits co-occurred 60.2% of the time (gray). The significant co-occurrence of genes associated with the utilization of positively associated carbon sources provides further support that biological properties contribute to positive trait associations. (PDF 521 kb)

Additional file 12: **Table S9.** Χ^2^ associations between gene presence and carbon traits. The “Same” column represents the number of times a gene was present and there was growth on the carbon source. The “Different” column represents the count of gene absence and growth plus gene presence and no growth. We corrected for multiple tests across associations with the Benjamini–Hochberg correction (*q* < 0.05 considered significant), which is represented by the column labeled “*p*_*Adj*_”. (XLSX 11 kb)

Additional file 13: **Table S10.** Species trait matrix, scored with growth (+), no growth (−), variable (*v*), or data unavailable (*n*), curated from *The Yeasts: A Taxonomic Study* [26]. (XLSX 245 kb)

Additional file 14: **Table S11.** Imputed binary species trait matrix. (XLSX 137 kb)

Additional file 15: **Table S12.** Proportion of correctly predicted traits across each method implemented to handle missing data. (XLSX 12 kb)

Additional file 16: **Table S13.** Newick file of D1/D2 sequences generated for 578 yeast species in RAxML-HPC BlackBox. (XLSX 23 kb)

## References

[1] Bleuven C, Landry CR (2016). Molecular and cellular bases of adaptation to a changing environment in microorganisms. Proc Biol Sci.

[2] Caspeta L, Nielsen J (2015). Thermotolerant yeast strains adapted by laboratory evolution show trade-off at ancestral temperatures and preadaptation to other stresses. MBio Am Soc Microbiol..

[3] Andersson DI, Jerlström-Hultqvist J, Näsvall J (2015). Evolution of new functions d*e novo* and from preexisting genes. Cold Spring Harb Perspect Biol..

[4] Patrick WM, Quandt EM, Swartzlander DB, Matsumura I (2007). Multicopy suppression underpins metabolic evolvability. Mol Biol Evol..

[5] Pavličev M, Cheverud JM (2015). Constraints evolve: context dependency of gene effects allows evolution of pleiotropy. Annu Rev Ecol Evol Syst Annual Reviews..

[6] Saltz JB, Hessel FC, Kelly MW (2017). Trait correlations in the genomics era. Trends Ecol Evol..

[7] Hosseini S-R, Wagner A (2016). The potential for non-adaptive origins of evolutionary innovations in central carbon metabolism. BMC Syst Biol..

[8] Gould SJ, Lewontin RC (1979). The spandrels of San Marco and the Panglossian paradigm: a critique of the adaptationist programme. Proc Biol Sci..

[9] True JR, Carroll SB (2002). Gene co-option in physiological and morphological evolution. Annu Rev Cell Dev Biol.

[10] Wiescher PT, Pearce-Duvet JMC, Feener DH (2012). Assembling an ant community: species functional traits reflect environmental filtering. Oecologia..

[11] Aspinwall MJ, Lowry DB, Taylor SH, Juenger TE, Hawkes CV, Johnson M-VV (2013). Genotypic variation in traits linked to climate and aboveground productivity in a widespread C4 grass: evidence for a functional trait syndrome. New Phytol..

[12] Tjoelker MG, Craine JM, Wedin D, Reich PB, Tilman D (2005). Linking leaf and root trait syndromes among 39 grassland and savannah species. New Phytol..

[13] Rosling A, Cox F, Cruz-Martinez K, Ihrmark K, Grelet G-A, Lindahl BD (2011). Archaeorhizomycetes: unearthing an ancient class of ubiquitous soil fungi. Science..

[14] Carnicer J, Barbeta A, Sperlich D, Coll M, Penuelas J (2013). Contrasting trait syndromes in angiosperms and conifers are associated with different responses of tree growth to temperature on a large scale. Front Plant Sci.

[15] Chapin FS, Autumn K, Pugnaire F (1993). Evolution of suites of traits in response to environmental stress. Am Nat..

[16] Larson G, Fuller DQ (2014). The evolution of animal domestication. Annu Rev Ecol Evol Syst..

[17] Wilkins AS, Wrangham RW, Fitch WT (2014). The “domestication syndrome” in mammals: a unified explanation based on neural crest cell behavior and genetics. Genetics..

[18] Fuller DQ, Denham T, Arroyo-Kalin M, Lucas L, Stevens CJ, Qin L (2014). Convergent evolution and parallelism in plant domestication revealed by an expanding archaeological record. Proc Natl Acad Sci..

[19] Lord J, Westoby M, Leishman M (1995). Seed size and phylogeny in six temperate floras: constraints, niche conservatism, and adaptation. Am Nat..

[20] Cadotte MW, Cavender-Bares J, Tilman D, Oakley TH (2009). Using phylogenetic, functional and trait diversity to understand patterns of plant community productivity. PLoS One..

[21] Marroig G, Cheverud JM (2001). A comparison of phenotypic variation and covariation patterns and the role of phylogeny, ecology, and ontogeny during cranial evolution of New World monkeys. Evolution..

[22] Ackerly DD, Dudley SA, Sultan SE, Schmitt J, Coleman JS, Linder CR (2000). The evolution of plant ecophysiological traits: recent advances and future directions new research addresses natural selection, genetic constraints, and the adaptive evolution of plant ecophysiological traits. Bioscience..

[23] Ghalambor CK, Walker JA, Reznick DN (2003). Multi-trait selection, adaptation, and constraints on the evolution of burst swimming performance. Integr Comp Biol..

[24] Roff DA, Fairbairn DJ (2007). The evolution of trade-offs: where are we?. J Evol Biol..

[25] Buckley LB, Davies TJ, Ackerly DD, Kraft NJB, Harrison SP, Anacker BL (2010). Phylogeny, niche conservatism and the latitudinal diversity gradient in mammals. Proc Biol Sci..

[26] Kurtzman C, Fell JW, Boekhout T (2011). The yeasts: a taxonomic study.

[27] Anderson PJ, McNeil K, Watson K. High-efficiency carbohydrate fermentation to ethanol at temperatures above 40 °C by *Kluyveromyces marxianus *var. *marxianus* isolated from sugar mills. Appl Environ Microbiol. 1986;51:1314–20. 10.1128/aem.51.6.1314-1320.1986 PMC239064 16347089

[28] Sylvester K, Wang Q-M, James B, Mendez R, Hulfachor AB, Hittinger CT (2015). Temperature and host preferences drive the diversification of *Saccharomyces* and other yeasts: A survey and the discovery of eight new yeast species. FEMS Yeast Res.

[29] Liti G, Carter DM, Moses AM, Warringer J, Parts L, James SA (2009). Population genomics of domestic and wild yeasts. Nature..

[30] Kaur R, Domergue R, Zupancic ML, Cormack BP (2005). A yeast by any other name: *Candida glabrata* and its interaction with the host. Curr Opin Microbiol..

[31] Messner R, Prillinger H, IBL M, Himmler G (1995). Sequences of ribosomal genes and internal transcribed spacers move three plant parasitic fungi, *Eremothecium ashbyi*, *Ashbya gossypii*, and *Nematospora coryli*, towards *Saccharomyces cerevisiae*. J Gen Appl Microbiol..

[32] Suh S-O, McHugh JV, Pollock DD, Blackwell M (2005). The beetle gut: a hyperdiverse source of novel yeasts. Mycol Res..

[33] Hong SG, Bae KS, Herzberg M, Titze A, Lachance MA (2003). *Candida kunwiensis* sp. nov., a yeast associated with flowers and bumblebees. Int J Syst Evol Microbiol..

[34] Sampaio JP, Gonçalves P (2008). Natural populations of *Saccharomyces kudriavzevii* in Portugal are associated with oak bark and are sympatric with *S. cerevisiae* and *S. paradoxus*. Appl Environ Microbiol..

[35] Legras J-L, Merdinoglu D, Cornuet J-M, Karst F (2007). Bread, beer and wine: *Saccharomyces cerevisiae* diversity reflects human history. Mol Ecol..

[36] Bergström A, Simpson JT, Salinas F, Barré B, Parts L, Zia A (2014). A high-definition view of functional genetic variation from natural yeast genomes. Mol Biol Evol..

[37] Borcard D, Legendre P, Drapeau P (1992). Partialling out the spatial component of ecological variation. Ecology..

[38] Fritz SA, Purvis A (2010). Selectivity in mammalian extinction risk and threat types: a new measure of phylogenetic signal strength in binary traits. Conserv Biol..

[39] Tristezza M, Tufariello M, Capozzi V, Spano G, Mita G, Grieco F (2016). The oenological potential of *Hanseniaspora uvarum* in simultaneous and sequential co-fermentation with *Saccharomyces cerevisiae* for industrial wine production. Front Microbiol..

[40] Sadineni V, Kondapalli N, Obulam VSR (2012). Effect of co-fermentation with *Saccharomyces cerevisiae* and *Torulaspora delbrueckii* or *Metschnikowia pulcherrima* on the aroma and sensory properties of mango wine. Ann Microbiol..

[41] Lachance M-A, Starmer WT (1986). The community concept and the problem of non-trivial characterization of yeast communities. Coenoses..

[42] Ramírez MA, Lorenz MC (2007). Mutations in alternative carbon utilization pathways in *Candida albicans* attenuate virulence and confer pleiotropic phenotypes. Eukaryot Cell..

[43] Lorenz MC. Carbon catabolite control in *Candida albicans*: new wrinkles in metabolism. MBio. 2013;4:e00034-13. 10.1128/mBio.00034-13 PMC3624514 23386434

[44] Brown AJP, Brown GD, Netea MG, Gow NAR (2014). Metabolism impacts upon *Candida* immunogenicity and pathogenicity at multiple levels. Trends Microbiol..

[45] Clauset A, Newman MEJ, Moore C (2004). Finding community structure in very large networks. Phys Rev E..

[46] Voordeckers K, Pougach K, Verstrepen KJ (2015). How do regulatory networks evolve and expand throughout evolution?. Curr Opin Biotechnol..

[47] Kanehisa M, Furumichi M, Tanabe M, Sato Y, Morishima K (2017). KEGG: New perspectives on genomes, pathways, diseases and drugs. Nucleic Acids Res..

[48] Xia T, Eiteman MA, Altman E (2012). Simultaneous utilization of glucose, xylose and arabinose in the presence of acetate by a consortium of *Escherichia coli* strains. Microb Cell Factories..

[49] Cadete RM, Heras AM, Sandström AG, Ferreira C, Gírio F, Gorwa-Grauslund M-F (2016). Exploring xylose metabolism in *Spathaspora* species: *XYL1.2* from *Spathaspora passalidarum* as the key for efficient anaerobic xylose fermentation in metabolic engineered *Saccharomyces cerevisiae*. Biotechnol Biofuels..

[50] Urbina H, Blackwell M (2012). Multilocus phylogenetic study of the *Scheffersomyces* yeast clade and characterization of the N-terminal region of xylose reductase gene. PLoS One..

[51] Wohlbach DJ, Kuo A, Sato TK, Potts KM, Salamov AA, LaButti KM (2011). Comparative genomics of xylose-fermenting fungi for enhanced biofuel production. Proc Natl Acad Sci..

[52] Hittinger CT, Rokas A, Bai F-Y, Boekhout T, Gonçalves P, Jeffries TW (2015). Genomics and the making of yeast biodiversity. Curr Opin Genet Dev..

[53] Haase MAB, Kominek J, Langdon QK, Kurtzman CP, Hittinger CT. Genome sequence and physiological analysis of *Yamadazyma laniorum* f.a. sp. nov. and a reevaluation of the apocryphal xylose fermentation of its sister species, *Candida tenuis*. FEMS Yeast Res. 2017;17:fox019. 10.1093/femsyr/fox019 PMC5418364 28419220

[54] Ayroles JF, Carbone MA, Stone EA, Jordan KW, Lyman RF, Magwire MM (2009). Systems genetics of complex traits in *Drosophila melanogaster*. Nat Genet..

[55] Shen X-X, Zhou X, Kominek J, Kurtzman CP, Hittinger CT, Rokas A (2016). Reconstructing the backbone of the Saccharomycotina yeast phylogeny using genome-scale data. G3 Genes|Genomes|Genetics.

[56] Hittinger CT, Rokas A, Carroll SB (2004). Parallel inactivation of multiple GAL pathway genes and ecological diversification in yeasts. Proc Natl Acad Sci..

[57] Voordeckers K, Brown CA, Vanneste K, van der Zande E, Voet A, Maere S (2012). Reconstruction of ancestral metabolic enzymes reveals molecular mechanisms underlying evolutionary innovation through gene duplication. PLoS Biol..

[58] Viigand K, Visnapuu T, Mardo K, Aasamets A, Alamäe T (2016). Maltase protein of *Ogataea* (*Hansenula*) *polymorpha* is a counterpart to the resurrected ancestor protein ancMALS of yeast maltases and isomaltases. Yeast..

[59] Mittelman K, Barkai N. The genetic requirements for pentose fermentation in budding yeast. G3 Genes|Genomes|Genetics. 2017;7:1743-52. 10.1534/g3.117.039610 PMC5473754 28404660

[60] Sato TK, Tremaine M, Parreiras LS, Hebert AS, Myers KS, Higbee AJ (2016). Directed evolution reveals unexpected epistatic interactions that alter metabolic regulation and enable anaerobic xylose use by *Saccharomyces cerevisiae*. PLoS Genet..

[61] dos Santos LV, Carazzolle MF, Nagamatsu ST, Sampaio NMV, Almeida LD, Pirolla RAS (2016). Unraveling the genetic basis of xylose consumption in engineered *Saccharomyces cerevisiae* strains. Sci Rep..

[62] Crespi BJ (2004). Vicious circles: positive feedback in major evolutionary and ecological transitions. Trends Ecol Evol..

[63] Kembel SW, Cowan PD, Helmus MR, Cornwell WK, Morlon H, Ackerly DD (2010). Picante: R tools for integrating phylogenies and ecology. Bioinformatics..

[64] Harrell Jr FE, Dupont MC. The Hmisc package. R Package, version 2.0-0. 2006;3:0–12.

[65] Suzuki R, Shimodaira H (2006). Pvclust: an R package for assessing the uncertainty in hierarchical clustering. Bioinformatics..

[66] Kurtzman CP, Robnett CJ (1998). Identification and phylogeny of ascomycetous yeasts from analysis of nuclear large subunit (26S) ribosomal DNA partial sequences. Antonie Van Leeuwenhoek..

[67] Kurtzman CP, Robnett CJ (2013). Relationships among genera of the Saccharomycotina (Ascomycota) from multigene phylogenetic analysis of type species. FEMS Yeast Res..

[68] Katoh K, Standley DM (2013). MAFFT multiple sequence alignment software, version 7: improvements in performance and usability. Mol Biol Evol..

[69] Miller MA, Pfeiffer W, Schwartz T. Creating the CIPRES Science Gateway for inference of large phylogenetic trees. Gateway Computing Environment Workshop (GCE). New Orleans: IEEE; 2010. p. 1–8.

[70] Stamatakis A (2014). RAxML version 8: a tool for phylogenetic analysis and post-analysis of large phylogenies. Bioinformatics..

[71] Orme D. The caper package: comparative analysis of phylogenetics and evolution in R. R Package, version 0.5.2. 2013;5:1–36.

[72] Gotelli NJ (2000). Null model analysis of species co-occurrence patterns. Ecology..

[73] Csárdi G, Nepusz T. The igraph software package for complex network research. Inter J Complex Syst. 2006:1695.

[74] Gancedo C, Flores C-L (2004). The importance of a functional trehalose biosynthetic pathway for the life of yeasts and fungi. FEMS Yeast Res..

