# Supplementary figures and images for: Factors driving metabolic diversity in the budding yeast subphylum
Source: BMC Biol. 2018 Mar 2;16:26. doi: 10.1186/s12915-018-0498-3 (PMC5833115; doi:10.1186/s12915-018-0498-3)

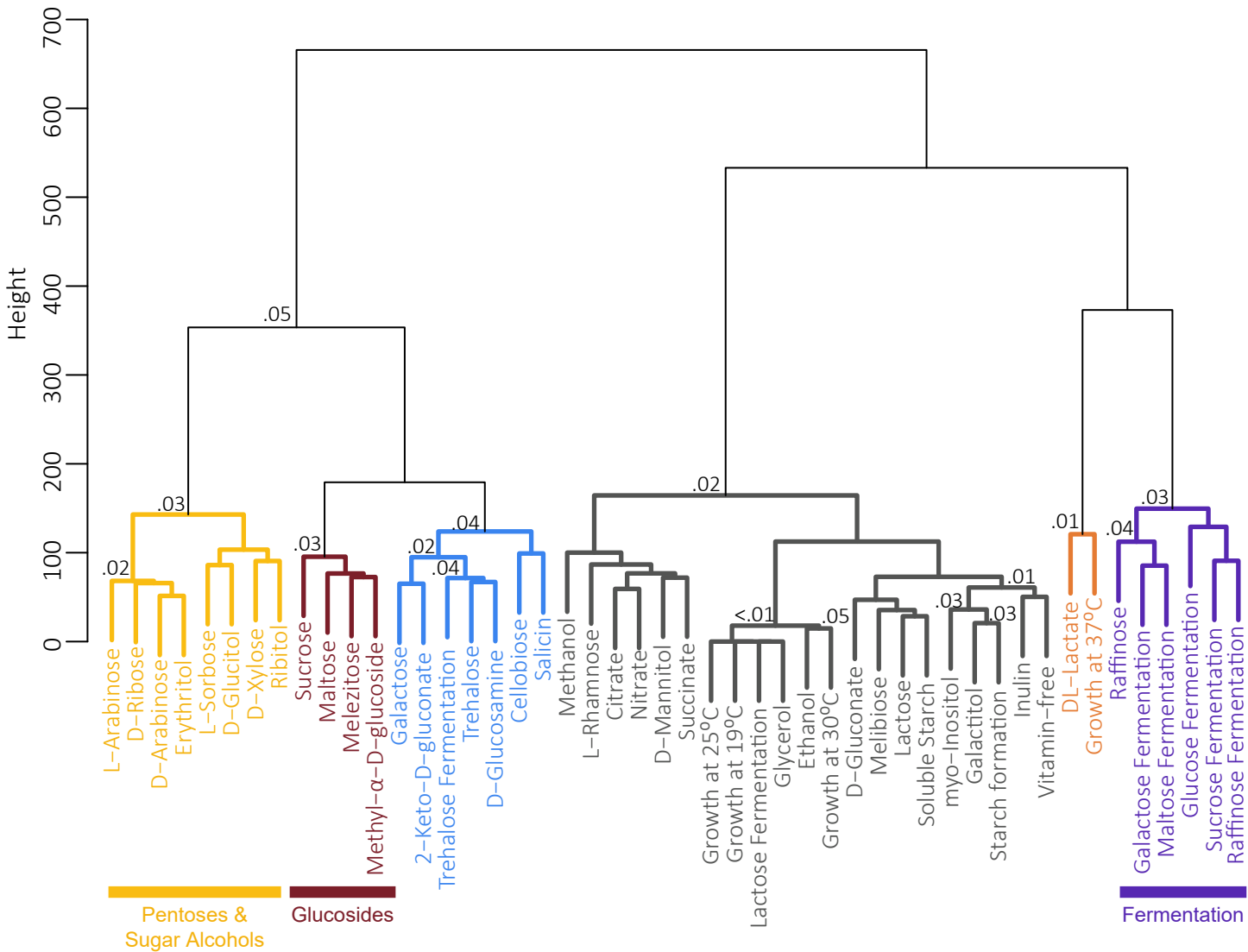

Supplement: Supplementary file 3 — Figure S1. Hierarchical cluster analysis of traits, using similarities among trait associations. The different colors represent selected significant clusters. Biologically meaningful clusters were named accordingly. Note that the strengths of the positive and negative associations with fermentation and with growth at high temperatures (37 °C) lead to clustering, which, in some cases, obscures the subtler associations among carbon source utilization traits that are readily observed in the network analysis presented in Fig. 6. (PDF 285 kb) [file 12915_2018_498_MOESM3_ESM.pdf]

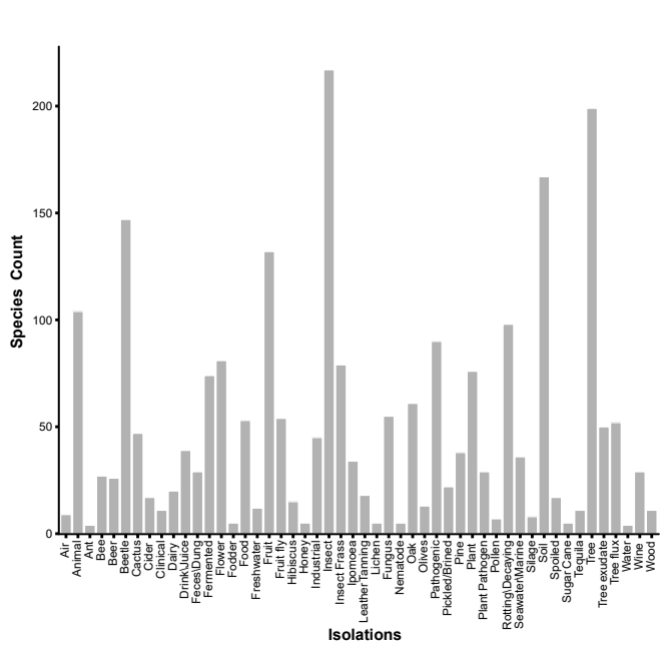

Supplement: Supplementary file 6 — Figure S2. Bar graph of number of species found in each isolation environment. (PDF 185 kb) [file 12915_2018_498_MOESM6_ESM.pdf]

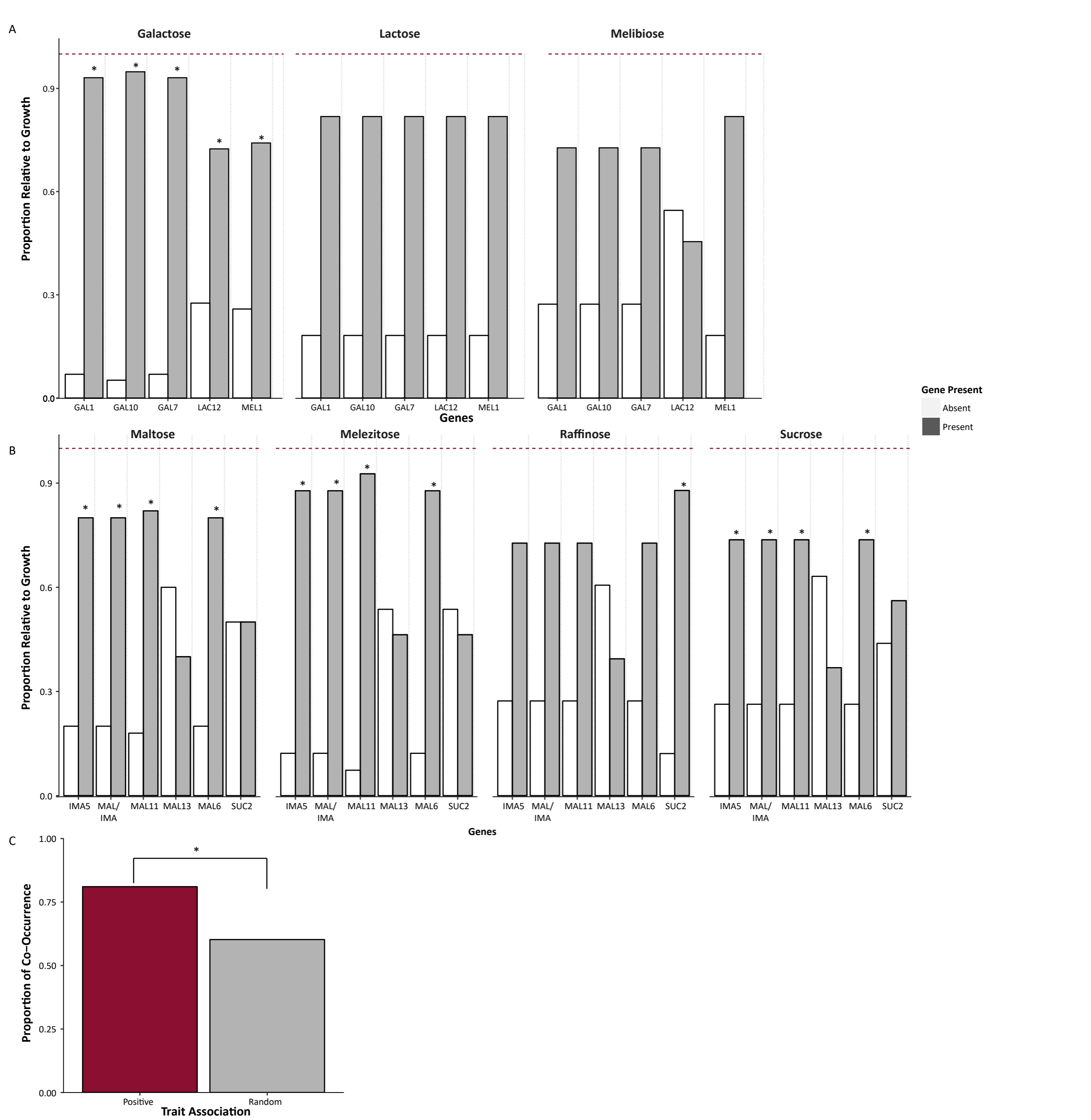

Supplement: Supplementary file 11 — Figure S3. Genes show pleiotropic functions for carbon utilization. We determined gene presence for 79 species for which we had trait data and curated genome sequences [55]. We quantified associations with gene presence and growth on two sets of carbon sources that show similar trait associations in Fig. 6. a Bar graph of growth on carbon sources (Contains Galactose) when genes are present (gray) or absent (white). Significant associations are denoted with an asterisk. It is well established that galactose utilization is associated with the presence of the GAL1, GAL7, and GAL10 genes [56]; this result serves as a control for our BLAST cutoffs. MEL1 and LAC12 are galactosidases that cleave the disaccharides melibiose and lactose, respectively, and were also significantly associated with galactose utilization across macroevolutionary timescales. The melibiose and lactose utilization data are consistent (but not significant) with this trend; however, there are few species that can use them. b Bar graph of growth on carbon sources (Glucosides) when associated genes are present (gray) or absent (white). We found significant associations (asterisk) between glucoside utilization genes and multiple carbon sources. These results suggest that pleiotropic genes could be responsible for the utilization of multiple carbon sources. c We quantified whether genes associated with traits that show positive trait associations co-occur more frequently than those that show random associations. Genes associated with positively associated traits (e.g., from a, GAL1, GAL7, GAL10, MEL1, and LAC12 comprise one set of genes, and from b, IMA5¸ IMA/MAL collapsed genes, MAL11, MAL13, MAL6, and SUC2 make up the second set) co-occurred 81.1% of the time (red), while genes associated with randomly associated traits co-occurred 60.2% of the time (gray). The significant co-occurrence of genes associated with the utilization of positively associated carbon sources provides further support that biologi [file 12915_2018_498_MOESM11_ESM.pdf]
